# Pharmaceutical Industry’s Engagement in the Global Equitable Distribution of COVID-19 Vaccines: Corporate Social Responsibility of EUL Vaccine Developers

**DOI:** 10.3390/vaccines9101183

**Published:** 2021-10-15

**Authors:** Meekang Sung, Yangmu Huang, Yuqi Duan, Fangjing Liu, Yinzi Jin, Zhijie Zheng

**Affiliations:** 1 College of Pharmacy, Seoul National University, 1, Gwanak-ro 38-gil, Seoul 08826, Korea; meekangs@snu.ac.kr; 2 Department of Global Health, School of Public Health, Peking University, 38 Xue Yuan Road, Beijing 100191, China; ymhuang@bjmu.edu.cn (Y.H.); 18811318259@163.com (Y.D.); liufangjing@pku.edu.cn (F.L.); zhengzj@bjmu.edu.cn (Z.Z.); 3 Institute for Global Health and Development, Peking University, Beijing 100871, China

**Keywords:** corporate social responsibility, COVID-19, vaccines, global health, COVAX, pharmaceutical companies

## Abstract

(1) Objectives: Inequality in the global distribution of COVID-19 vaccines has brought about great challenges in terms of resolving the pandemic. Although vaccine manufacturers are undoubtedly some of the most influential players, studies on their role in global vaccine distribution have been scarce. This study examined whether the pharmaceutical industry is acting according to the principles of corporate social responsibility (CSR) during the pandemic. (2) Methods: Three categories were used to analyze the CSR of vaccine developers. The first was research and development: effectiveness, funding, and profits were measured. The second was transparency and accountability: the transparency of clinical trials and vaccine contracts was analyzed. The final was vaccine delivery: the status of the provision of vaccines to COVAX and lower-income countries, intellectual property management, manufacturing agreements, and equitable pricing were measured. (3) Results: Vaccine developers have acquired large profits. The vaccine delivery category faces the most challenges. Participation of pharmaceutical companies through COVAX was significantly low, and most vaccine supply agreements were secretive, bilateral deals. It was not clear if companies were maintaining equitable pricing. The evaluation indicated that the companies’ CSR practices have differed during the pandemic. (4) Conclusions: Our study contributes to the methodology of assessing the CSR of vaccine developers. This would help understand the current COVID-19 vaccine distribution inequality and propose that pharmaceutical companies re-examine their roles and social responsibilities.

## 1. Introduction

The coronavirus disease 2019 (COVID-19) pandemic has tremendously affected individuals’ health and global stability. As of 9 September 2021, there have been 222,406,582 confirmed cases, including 4,592,934 deaths globally (WHO, 2021). As new variants of the virus continue to appear, people are increasingly starting to think that because it might be impossible to completely eradicate the virus, people should learn how to “live with it” [1]. Nevertheless, the development and distribution of vaccines are perceived as an indispensable solution to end or cope with the pandemic. Many countries and pharmaceutical companies have engaged at an unprecedented speed in developing various vaccines, and the vaccine roll-out started earlier than expected. Behind this good news lies the shadow of the inequality in the global distribution of COVID-19 vaccines.

To tackle this issue, the COVAX initiative was launched in April 2020, with the aim of accelerating the development, allocation, and equitable access to COVID-19 vaccines through a global collaboration between governments, intergovernmental organizations, and private sectors. COVAX acts as a platform to support the research, development, and manufacturing of a wide range of COVID-19 vaccine candidates and negotiate their pricing [2]. There are two possible arrangements to participate in COVAX; the “committed purchase” arrangement, and the “optional purchase” arrangement.

For most low- and middle-income countries (LMICs), COVAX is the only viable way to access vaccines. It is also useful to several higher-income self-financing countries that have no bilateral deals with developers [3]. However, COVAX is having a hard time procuring enough vaccines to distribute internationally, especially LMICs. As a result, the delivery of vaccines is being significantly delayed. To make matters worse, recently, several high-income countries have prepared to offer their citizens a third COVID-19 vaccine as a booster shot. This was criticized by the WHO Director-General Tedros Adhanom Ghebreyesus, because this could aggravate the situation of many countries suffering from vaccine shortages. He further called for countries to wait until 2022 to start offering booster shots [4].

The global problem of COVID-19 vaccine distribution is related to many stakeholders: the state governments, various companies, communities, NGOs, just to list a few. There has been criticism about “vaccine nationalism” [5] and inequality associated with the distribution of COVID-19 vaccines. There are also reports on the issue of transparency of clinical trials [6], global supply, contracts, and prices [7,8]. However, although vaccine manufacturers are undoubtedly one of the most influential players, studies on their role in the global vaccine distribution landscape have been scarce. Therefore, further study on the role of vaccine manufacturers is much needed to improve our understanding of the companies’ decisions and thus improve inequality in vaccine distribution. Our study tried to address this evidence gap, integrating the concept of CSR to explore the ethical responsibilities and the current role of the pharmaceutical companies in the COVID-19 situation.

The specific research questions are as follows: (1) Have the vaccine developers considered CSR enough during COVID-19, or were they mainly focusing on short-term returns?; (2) How could the CSR activity of pharmaceutical companies in a unique pandemic situation be measured?; (3) Was there consistency with the companies’ existing CSR practice and their actions during the pandemic?

## 2. Theory and Literature Review

### 2.1. Definition and Motivations of CSR

CSR is defined by the International Organization for Standardization as a way in which companies can address and manage social, economic, and/or environmental issues for the benefit of communities and as involving actions related to human rights, social inclusion, and other related concerns [9]. Generally, CSR is referred to as social responsibility more often than CSR [10]. Dirk Matten and Jeremy Moon presented a definition of CSR as the “policies and practices of corporations that reflect business responsibility for some of the wider societal good. Yet the precise manifestation and direction of the responsibility lie at the discretion of the corporation” [11].

There are broadly a couple of reasons why companies practice CSR: (1) CSR as a business strategy; and (2) CSR as an ethical responsibility. First, CSR could be an excellent ‘win–win’ strategic management tool for accomplishing sustainable development [12]. Resnik points out that social responsibilities make good business sense because they are closely related to the companies’ reputations [13]. In this perspective, CSR is utilized to manage various stakeholders, who can significantly influence the companies’ performance. The stakeholders include shareholders, business partners, employees, suppliers, customers, local communities, non-government organizations (NGOs), government officials (GOs), and the environment [14]. Previous studies have suggested that corporate social sustainability can simultaneously protect companies from boycotts and other actions by different stakeholders, and thus reduces the risk of litigation and financial difficulties [15].

Among these stakeholders, the most important for companies are employees (an internal stakeholder group), and consumers and communities (two distinct external stakeholder groups [16]) According to which stakeholder they address, CSR can be categorized into internal and external. Internal CSR refers to “formal CSR initiatives within which employees can participate and reap developmental benefits which show employers’ respect to their employees”. and external CSR refers to “the practices focused on stewardship toward the local community, the natural environment, and consumers” [17]. The external “community” stakeholder can have various traits and are related to the firm’s supply chain across the globe [18]. Idemudia views that the company and community are interconnected [19], and Kochhar argues that they can affect each other’s decisions [20]. Community pressure can also influence a firm’s CSR policy and its implementation strategies [21]. COVID-19 vaccine distribution is related to the global community (external stakeholder group) much more broadly and greatly than the employees of the companies (internal stakeholder group); therefore, our paper focuses on external CSR.

Secondly, CSR can also be viewed as an ethical responsibility of companies. Resnik argues that because corporations make decisions that have an important impact on humanity, they should be considered moral agents, and therefore have social responsibilities [22]. Ethical responsibilities embody the full scope of norms, standards, values, and expectations that reflect what consumers, employees, shareholders, the community, and other stakeholders regard as fair, just, and consistent, with respect for and the protection of stakeholders’ moral interests and rights [22]. Ethical CSR practices are morally obligatory and are strongly related to the responsibilities that companies have with those individuals or groups to whom they may cause damages during their business activity. These responsibilities exceed those established in the legislative framework. Examples of ethical CSR are altruistic or philanthropic CSR practices that are related to a utilitarian perspective—equity, justice, and social care—without necessarily benefiting the company’s economic and financial situation [23].

During a crisis, CSR and the responsibility and accountability of a company to its stakeholders are especially important [24]. The COVID-19 pandemic represents one of the most impactful shocks worldwide [25,26]. Its direct and indirect effects expose both the companies and community to extraordinary external forces. As many stakeholders become more vulnerable, companies can be tempted to make short-term decisions trying to make a quick recovery or make immediate profits that guarantee their survival, limiting the funds allocated to CSR [27]. However, such approaches would likely harm the long-term trust of stakeholders in the companies and negatively influence the business management of the company. More importantly, it is contrary to the ethical responsibility they have.

Thus, with the prescriptions of CSR theory, in this study, we propose that corporations, especially vaccine developers, should support society and its vital stakeholders during the COVID-19 pandemic. Several studies have focused on CSR during COVID-19. Mahmud et al., explored the U.S. CSR leaders’ CSR initiatives to the COVID-19 pandemic using the stakeholder theory [12]. García-Sánchez et al., analyzed the involvement of Spanish companies’ CSR during the COVID-19 epidemic [24]. However, no study has yet specifically explored the CSR of pharmaceutical companies during the COVID-19 pandemic, which leads us to the next topic—how was CSR practiced in pharmaceutical companies generally?

### 2.2. CSR in Pharmaceutical Companies

CSR is particularly important for pharmaceutical companies. The products and services they provide are strongly related to the health of both individuals and the entire society. Hurst utilized the 2005 United Nations Educational, Scientific, and Cultural Organization’s Universal Declaration on Bioethics and Human Rights (UDBHR) to establish ethical reasoning regarding the societal responsibilities of pharmaceutical companies [28]. The author specifically concentrated on Article 14 of the UDBHR—social responsibility and health. Article 14 places a moral responsibility upon private enterprises to contribute to the promotion of health and social development for pharmaceutical and biotechnology companies; it concerns adequate pricing, access to quality health care, and essential medicines [28].

Common CSR activities of pharmaceutical companies include differential pharmaceutical pricing, strengthening drug deployment infrastructure in LMICs, and targeted research and development for neglected diseases. Martínez-Palomo argues that a socially responsible company should consider access to treatment for LMICs and should have the following five priorities: (1) pricing; (2) patent; (3) joint public–private initiatives; (4) research and development; and (5) the appropriate use of drugs [28]. Vaccine pricing has historically received considerable attention, with studies highlighting its role in generating sustainable access in LMICs [29]. Price differentiation is a promising approach for access to medications. In such a schema, high-income countries (HICs) would pay more for drugs, whereas LMICs would only pay production costs and not the cost of research and development. Therefore, prices are set in a way that reflects a country’s ability to pay, as measured by the level of income [30]. The difference in prices in different markets is referred to as “tiered pricing”, a term which emphasizes the perspective of the producer [31]. “Equity Pricing”, or “equitable pricing”, emphasizes the perspective of the consumer, and whether a price is affordable and acceptable. Although tiered pricing may lead to equitable prices, the concepts are not equivalent and tiered prices may not be affordable.

Droppert and Bennett have explored the motives of pharmaceutical companies engaging in CSR activities [32]. They do so with motives of reputational benefit, employee satisfaction, higher rankings in sustainability indices, entrance into new markets, long-term economic returns, and improved population health. We can see that CSR activities are not just about obligation, but that such activities are positively related to the actual economic performance of the company. Min et al. studied the relationship between CSR and the performance of companies in the pharmaceutical industry, and their results show that the adoption of responsible social behavior positively influences the economic profits of companies [33].

There is a long history of criticism towards pharmaceutical companies for not properly engaging in CSR [34], but there has been considerable pressure from the international society, and companies have made efforts to improve it. For example, UNESCO, agreeing with the assessments made by the non-governmental organizations (NGOs) Oxfam, Save the Children, and Voluntary Service Overseas, challenged the pharmaceutical industry to adopt a broader scope of responsibility and improve its efforts to tackle the health crisis affecting LMICs [35]. As a response, many pharmaceutical companies have recently adopted price differentiation policies [36].

Some studies give credit to pharmaceutical companies’ philanthropic activities [37]. For example, in 1987, Merck took the Mectizan Donation initiative to arrange the free drug distribution in collaboration with the WHO, the World Bank, and their other partners. GlaxoSmithKline have also donated vast quantities of medicines in conjunction with the WHO and other partners to eliminate lymphatic filariasis. Novartis regularly donates medicines as a part of CSR programs to abolish leprosy [38].

### 2.3. Measuring CSR

Several indexes have been developed to measure the CSR of pharmaceutical companies. For example, Gruskin, S. and Raad, Z. assessed drug companies using top-down, bottom-up, and horizontal approaches [37]. The Dow Jones Sustainability Index evaluates the sustainability performance of thousands of companies, including pharmaceuticals [39]. The most widely used and comprehensive index is the Access to Medicine Index [36], developed by The Access to Medicine Foundation. The index measures how the world’s 20 largest pharmaceutical companies are addressing access to medicine in 106 LMICs for 82 diseases, conditions, and pathogens. However, they focus on CSR activities on a general scope, and the 20 pharmaceutical companies measured do not include Moderna, Sinopharm, and Sinovac. Therefore, the index has limitations in understanding the CSR of the important vaccine developers in an unprecedented pandemic.

The Access to Medicine Foundation also developed the Access to Vaccines Index, which measures what vaccine manufacturers and developers are doing to help improve the global immunization coverage. Eight companies were evaluated in research and development, pricing and registration, and manufacturing and supply [36]. This index is also not sufficient to understand the current situation, because the last version was published in 2017 and the COVID-19 vaccine is not the same as the other vaccines. There are novel technologies associated with the manufacturing of the vaccine, and the developed countries are still far from recovering from the strike of COVID-19, which calls for “vaccine nationalism” in acting. This leads to different company attitudes on vaccine distribution.

Additionally, many ESG (Environmental, Social, and Governance) scores have been used in the evaluation of the companies. There are dozens of rating providers—MSCI, Sustainalytics, RepRisk, Bloomberg, and new-entrant Institutional Shareholder Services (ISSs) currently dominate the market [40]. For example, the MSCI ESG Ratings evaluate corporate behavior, product safety and quality, corporate governance, toxic emissions and waste, human capital development, and access to health care for the pharmaceutical industry [41]. They provide ratings for all companies in our research scope, except Sinovac, and the score is as follows: Pfizer (B), AstraZeneca (AA), Johnson & Johnson (BBB), Moderna (BB), and Sinopharm (BB). The ratings are also too broad to specifically understand the pharmaceutical companies’ role in vaccine distribution.

Thus, in this study, we attempted to develop a new method to measure the CSR of pharmaceutical companies in such a unique global situation. Due to restrictions of time and resources, among the pharmaceutical industry, we focused on the vaccine developers which are directly involved in the distribution of COVID-19 vaccines.

## 3. Materials and Methods

This study analyzed pharmaceutical companies which have announced that their COVID-19 vaccines have adequate clinical trial data and therefore have successfully started their worldwide vaccine roll-out. Specifically, we focused on the six vaccine developers that have passed the Emergency Use Listing Procedure (EUL) of the World Health Organization (WHO) by August 2021: Pfizer/BioNTech, AstraZeneca–Oxford, Janssen, Moderna, Sinopharm, and Sinovac. The AZD1222 Vaxzevria and Covishield vaccine is listed as a separate developer (AstraZeneca–Oxford and Serum Institute of India) in the status of COVID-19 vaccines within the WHO EUL/PQ evaluation process. These two vaccines were both developed by AstraZeneca–Oxford, only with a different manufacturing site; therefore, we analyzed them together as a same vaccine developer (AstraZeneca–Oxford). Additionally, because only the Sinopharm/BIBP has finalized the EUL procedure and WIBP has not, we only included Sinopharm/BIBP in our analysis. Details of the EUL-listed vaccines we included in the study are presented in Supplementary Materials Table S1.

EUL is a risk-based procedure for assessing the suitability of unlicensed vaccines, therapeutics, and in vitro diagnostics [42]. The EUL pathway involves a rigorous assessment of late phase II and phase III clinical trial data as well as substantial additional data on safety, efficacy, quality, and a risk management plan, with a focus on the needs of LMICs. Therefore, we assumed that the vaccines which had passed EUL passed the minimum reliability to be used worldwide.

This study modified the Access to Medicine Index to measure CSR in pharmaceutical companies during the pandemic (Supplementary Materials Table S2). The Access to Medicine Index uses 33 indicators grouped into 3 technical categories [43]. First, the Governance of Access category looks at how companies govern, plan for, and manage the achievement of access-linked objectives, ensuring that they apply processes that minimize the risk of non-compliant or corrupt behavior occurring. However, the indexes in this category address long-term governance and broad corporate initiatives. The COVID-19 pandemic situation is a rather short-term event; therefore, we did not integrate the Government of Access indicators.

Second, the Research and Development category evaluates the pharmaceutical companies’ activities to develop or adapt products, and in response to the needs of people living in LMICs [35]. We included the Research and Development category, and specifically analyzed the effectiveness of the vaccines, storage, and manufacturing capacity. Although the information of characteristics of vaccines such as the storage conditions and manufacturing capacity is essential in understanding the vaccine distribution to LMICs, it is not in the scope of CSR. Therefore, we presented all the information but only used assessments on the effectiveness of the vaccines as an indicator of CSR activity.

We also analyzed the amount of funding the companies received. It is vital to understand the funding and profits of the firms to evaluate whether the companies were really ‘risk-taking’ and proactive in vaccine product development and the building of R&D capacity. This especially applies to the current pandemic, because the vaccine drive was funded extensively by many governments and public organizations. The profits companies have generated from the COVID-19 vaccine were also included. Although companies had faced huge opportunity costs, and profits are the main reason why companies exist especially for private-owned companies, their actions during the pandemic might be different. In this paper, we are not proposing that companies should not pursue profit, but used their stance on COVID-19 vaccine profits to evaluate their extent of CSR.

Thirdly, the Product Delivery category measures companies’ post-development actions to ensure they offer equitable access to their products. There are three main access strategies: equitable pricing, responsible intellectual property management, and product donations [44]. This category was used in this study for the evaluation of CSR activities regarding COVID-19 vaccines. The correlation coefficient between GDP per capita and vaccine prices was utilized to analyze the scope of equitable pricing. To measure the extent to which companies are trying to ensure equitable access, we calculated the percentages of their products they are giving to COVAX and LMICs. The companies’ status regarding IP management, manufacturing agreements, and equitable pricing was also considered.

Our study further introduced the Transparency and Accountability category. Not only is transparency and the accountability of information closely related to the trust and reliability of a company, but it is also critical in tackling the gap of weak bargaining power [45]. This category is particularly important during a crisis, when the responsibility and accountability of a company are especially important in CSR [24]. In addition, the rushed development process and the many emergency qualifications and listings made for COVID-19 may have affected the transparency and accountability of the firms. Therefore, we propose that this category is crucial as an index to measure CSR during the COVID-19 pandemic. We analyzed transparency in two dimensions: clinical trials and vaccine supply agreements.

Based on the findings according to each category, we calculated the score of the EUL vaccine developers’ CSR activity during the COVID-19 pandemic as follows:

1. Research and Development
  a. Effectiveness: 1 point for over 90% effectiveness and consistent clinical trial results;
  b. Profits: 1 point for stating non-profit on vaccine sales.
2. Transparency and Accountability
  a. Clinical trials: 1 point for publicly sharing clinical trial protocols;
  b. Vaccine Contracts: 1 point for publishing opening over 20% of vaccine price data to public.
3. Product Delivery
  a. COVAX %: 1 point for providing more than 20% of vaccine supplies to COVAX;
  b. Distribution to Lower-Income Countries: 1 point for distributing more than 40% of vaccine supplies to countries other than HICs;
  c. Equitable pricing: 1 point for a positive correlation coefficient between GDP per capita and vaccine prices;
  d. Intellectual Property Strategy: 1 point for agreeing to waive IP rights during the COVID-19 pandemic;
  e. Manufacturing agreements: 1 point for actively participating in manufacturing agreements with various countries, including lower-income countries; more than 15% of manufacturing agreements (including CDMO and Technology Transfer) are made with countries other than high-income countries.

Data from January 2020 to August 2021 were acquired for the analysis. The sources of data for Research and Development, Transparency and Accountability, and Product Delivery were from financial reports, company announcements, credible news materials, and reports from international organizations such as the United Nations Children’s Emergency Fund (UNICEF) and the World Health Organization. Data on the number of secured vaccine doses, contracts, and vaccine prices were obtained by the UNICEF COVID-19 Vaccine Market Dashboard from January 2020 to August 2021 [7]. This is the most comprehensive database on COVID-19 vaccine contracts, and it uses secondary sources such as media reports and press statements. The selection of databases and calculation of the results were conducted independently by S.M., and J.Y. cross-checked the credibility and accuracy. All specific sources are inserted as citations in the tables in the Results. Explanations on the type of the sources and search methodologies are presented in Supplementary Materials Table S3.

## 4. Results

### 4.1. Research and Development

#### 4.1.1. Effectiveness, Storage, and Manufacturing Capacity

Among the six EUL vaccines, mRNA vaccines (Pfizer/BioNTech and Moderna) appeared to have the highest effectiveness. Other vaccines using traditional techniques showed quite large variations in effectiveness. (Table 1) The storage condition of the mRNA vaccines is comparatively delicate, and it might be tricky for the vaccine to survive through cold chains during the delivery process. Therefore, it could be less practical for hard-to-reach parts of the world than other vaccines that can simply be refrigerated.

Pfizer/BioNTech and AstraZeneca–Oxford were found to have the highest manufacturing capacity and are providing the greatest number of vaccines globally (Table 1). However, we must acknowledge that the manufacturing capacity announced by the companies may be overstated. According to the Monetary Fund in Washington DC, although the pharmaceutical industry expects to have made a total of about ten billion vaccine doses by the end of 2021, the industry is likely to have produced only around six billion doses by the end of the year [46].

#### 4.1.2. Profit Generation

Table 2 presents the amount of funding the vaccine developers received, the revenues they made from it, and the stance on profits of the vaccine developers. We acquired funding data from The Knowledge Network on Innovation and Access to Medicines (published in May 2021) [47]. Funder type includes CEPI, the public sector, the private sector, and philanthropic organizations. We further included data which integrates Advance Purchase Agreements (APAs) as funding, because these act as incentives for development. When APAs are considered, Pfizer/BioNTech has received the largest amount of funding, followed by Moderna and Janssen. When APAs were not considered, Janssen has received the largest amount of funding, followed by Moderna and Pfizer. Revenue data were acquired from the financial reports or SEC filings of each company.

Pfizer/BioNTech had been reported to be making considerable profits from the vaccine, reaching nearly one-quarter of its total revenue in the first quarter of 2021. Pfizer/BioNTech further expected its vaccine to generate USD 33.5 billion in revenue this year, up from its previous estimate of USD 15 billion [48]. Moderna is also expected to add a huge number of sales this year. It has reported a total of USD 5.9 billion generated by product sales of the COVID-19 vaccine. AstraZeneca–Oxford reported USD 1169 million of revenue from the vaccine, which accounts for 7% of total revenue. Janssen reported vaccine sales revenue was USD 100 million at Q1, and 164 million USD at Q2, adding up to 264 million USD for the first six months. Sinopharm and Sinovac did not make public announcements or shared profit objectives. Sinopharm reported a total of RMB 157,494,669 (about USD 24.4 billion) of revenue from the “pharmaceutical distributions” sector, but the report did not show the specific amount of profits generated from the COVID-19 vaccines [49]. There were no financial reports of 2021 available from Sinovac. A Bloomberg article estimated that Sinovac could earn as much as USD 25 billion, and Sinopharm as much as USD 23 billion by COVID-19 vaccine sales this year [50].

The companies making the novel mRNA vaccine, Pfizer/BioNTech and Moderna, were anticipating some returns. Pfizer CEO Albert Bourla said the company “will make a very, very marginal profit” in a *Time* interview [51]. Moderna’s CEO, Stéphane Bancel, told *Business Insider* that “they will not charge too high a price” [52]. In contrast, AstraZeneca–Oxford and Janssen pledged to not make profits on the COVID-19 vaccine [53,54].

**Table 2:** Funding, revenues, and stance on the profit generation of EUL COVID-19 vaccines.

	Pfizer/BioNTech	AstraZeneca–Oxford	Janssen	Moderna	Sinopharm	Sinovac
Funding ^1^ (APA included) (million USD)	18,549	4967	5928	8337	145	515
Funding ^2^ (APA excluded) (million USD)	800	115	1028	955	145	515
Revenues from vaccine sales in 2021 (Q1–Q2 this year, billion USD)	11.3 [48,55]	1.2 [56]	0.3 [57,58]	5.9 [59,60]	- ^3^	-
Announcements	“marginal profit” [51]	no profit [54]	no profit [52]	“will not charge too high a price” [53]	-	-

Notes: Data on funding were acquired from COVID-19 Vaccines R&D Investments [47] (published May 2021). ^1^ Funder type includes CEPI, the public sector, the private sector, and philanthropic organizations, and Advance Purchase Agreements. ^2^ Funder type includes CEPI, the public sector, the private sector, and philanthropic organizations. Revenue data were acquired from financial reports of companies. ^3^ “-” indicates that there have been no public announcements. Companies’ stances on profit generation were searched on credible news articles and company announcements [52,53,54,55].

### 4.2. Transparency and Accountability

#### 4.2.1. Clinical Trials

The subject of this study was vaccines which passed the EUL, it guarantees a certain degree of transparency in clinical results. However, the transparency and accountability of the vaccines were not without problems. Analysis of the registered clinical trials for the top vaccines revealed that results from just 45% of these trials had been announced. Clinical trial protocols had been published for just 12% of trials [6].

Table 3 summarizes the transparency of the clinical trial protocols, results, and published contracts. Pfizer/BioNTech, AstraZeneca–Oxford, Janssen, Moderna, and Sinovac had announced their clinical trial protocol publicly, thus increasing international credibility. Sinopharm have not shared their clinical trial protocols publicly. Although there were an overall lack of peer-reviewed clinical data results for Sinopharm, it should be considered that not long a time has passed since the clinical trials for vaccines began.

Even among the published clinical trials, there was some criticism. Janssen’s efficacy numbers were hard to grasp, with separate numbers for different age groups [6]. In November 2020, AstraZeneca–Oxford announced preliminary results of its phase III trial by a press release which created confusion due to differences in dosing, where a 15% lower dose led to a nearly 30% higher efficacy rate [61]. Further explanatory details were privately shared with industry experts and selected media.

#### 4.2.2. Vaccine Contracts

Overall, there was a lack of disclosure of the federal contracts with companies. Only 6% of the concluded agreements were formally published on 5 March 2021 and only 18.6% of vaccine prices were known to public of the until August 2021. Additionally, because most of the vaccine supply agreements were bilateral deals, the details of the contract between the pharmaceutical company and the governments were rarely revealed. Of the handful of contracts that had been published, almost all include significant redactions of key information such as total price paid, price per dose, and delivery schedules [6]. Just one contract was published without redactions.

### 4.3. Product Delivery

#### 4.3.1. Percentage of Vaccines in COVAX Vaccine Supply Agreements

Table 4 analyzed how the procured vaccines were distribute using two categories: (i) type of agreement (multilateral, bilateral) and (ii) country income level. The type of agreement was classified as multilateral and bilateral. Multilateral deals include contracts with COVAX, the European Commission, and the African Union. Country income level was classified as HIC (high-income countries), UMIC (upper-middle-income countries), LMIC (lower middle-income countries), and LIC (low-income countries). The countries were classified according to the World Bank country classifications by income level: 2020–2021 [62]. Data on the number of secured vaccine doses and contracts were obtained by the UNICEF COVID-19 Vaccine Market Dashboard until August 2021.

We found that bilateral deals were dominant (62.09%) in vaccine supply agreements. The vaccine developers occasionally made multilateral deals with COVAX, the European Commission, and the African Union. Thus far, Moderna has supplied their products via COVAX with the highest rate (26.89%). Sinovac supplied their products via COVAX with the lowest rate (7.7%), followed by Pfizer/BioNTech (13.4%). Although Pfizer agreed to provide the U.S. government 500 million doses for the use of COVAX on 10 June 2021, it is not a multilateral deal through the facility, and therefore may undermine the bargaining power of COVAX. Sinopharm and Sinovac recently agreed to supply to COVAX, on 12 July 2021 [63].

#### 4.3.2. Distribution to Lower-Income Countries

The shortage of supplies for lower-income countries had been highly controversial worldwide. According to the United Nations press release, although more than 700 million vaccine doses have been administered globally, richer countries have received more than 87%, and low-income countries received just 0.2% [64]. Our results (Table 4) are consistent with the concerning reports. Most vaccine agreements of EUL COVID-19 developers are made with HICs (49.64%), whereas LMICs are not able to secure enough doses (18.97%), and LICs have barely succeeded in securing any vaccine shots (0.1%). This was especially highlighted in the cases of Pfizer/BioNTech and Moderna; most of their vaccines were secured by HICs (66.87% and 69.75%, respectively).

There was a conspicuous discrepancy between the distributed vaccine doses and the population of countries. The population in HICs was 1.25 billion in 2020, constituting 16% of the global population (7.753 billion), and they procure 49.6% of the vaccines; the population in UMICs was 2.514 billion in 2020, constituting 43% of the global population, but only secured 19% of the vaccines (not counting COVAX); the population in LMICs was 3.331 billion in 2020, 32% of the global population, and secured 13% of the vaccines (not counting COVAX); the population in LICs was 665 million in 2020, about 8% of the global population, but barely received any doses of the COVID-19 vaccine. (Population data are from the World Bank.)

#### 4.3.3. Equitable Pricing

We calculated the average price the vaccine developers have agreed to provide their products to HICs, UMICs, LMICs, and LICs (Table 5). Data on the number of secured vaccine doses, contracts, and vaccine prices were obtained by the UNICEF COVID-19 Vaccine Market Dashboard [7] up to August 2021.

**Figure 1:**
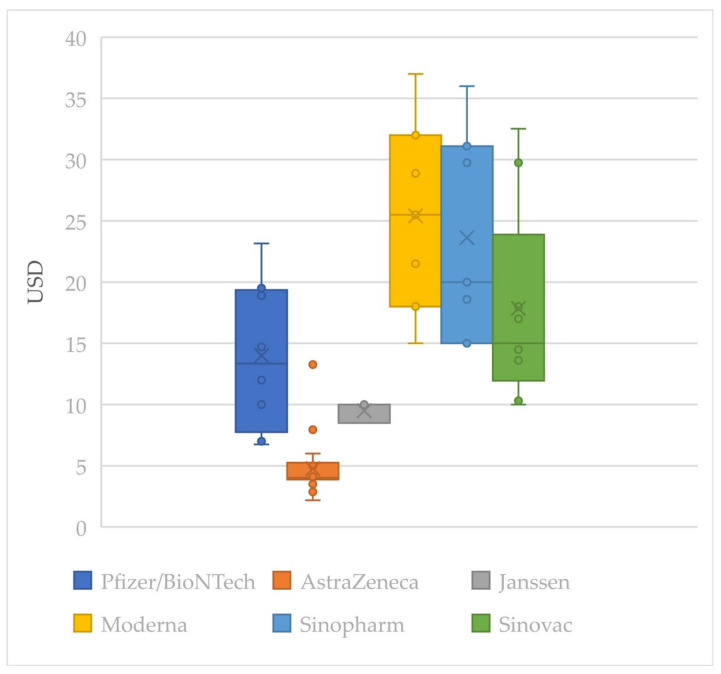
Box-and-whisker diagram of vaccine prices by company.

Of the total 279 vaccine supply agreements, only 56 vaccine prices are known. The known vaccine prices are presented in Figure 1 as a box-and-whisker diagram. None of the vaccine prices given to LICs were available. AstraZeneca–Oxford and Janssen vaccines were available at relatively low prices. However, the price variation of the vaccines was large; thus, it was hard to compare the vaccine’s price by the average price. All the prices determined by a bilateral deal between vaccine developers and the federal government were much higher than the price COVAX guarantees for its participants (USD 3.2/dose).

In an equitable pricing system, it is expected that prices would broadly increase as a buyer’s ability to afford it increases. However, the data indicate that this may not be the case. There is not much of a price difference between HICs (USD 16.41) and UMICs (USD 15.02); AstraZeneca–Oxford had a higher average price for UMICs than HICs. Apparent price differentiation according to the nation’s economic ability also could not be found.

We calculated the correlation coefficient between GDP per capita and the vaccine prices to find out if each vaccine developer was practicing equitable pricing. The correlation coefficient was calculated with the ‘CORREL’ function in Excel. As a result, Pfizer/BioNTech was found to have the highest correlation coefficient (0.82), suggesting that they priced their products according to the principles of equitable pricing. Sinopharm and Sinovac were also providing vaccines at a more affordable price to lower-income countries. However, AstraZeneca–Oxford does not seem to have made their products cheaper to lower-income countries, and the coefficient was even calculated as negative. This result may have occurred because AstraZeneca–Oxford is one of the cheapest vaccines, and the price was quite affordable even to lower-income countries. There was very little available vaccine price data for Janssen (only 3). More information about the contracts needs to be revealed for accurate analysis.

#### 4.3.4. Intellectual Property Strategy

There is an ongoing debate about whether the World Trade Organization should approve a proposal from India and South Africa which would temporarily suspend the enforcement of global intellectual property rules. It is being backed by more than 100 countries, along with international organizations including the World Health Organization and the United Nations AIDS charity, UNAIDS. These countries argued that doing so would unlock key vaccine formulas, boost global production, and improve access [65]. Russia, China, and most recently, the United States, have decided to back up the waiving of intellectual property rights [66].

The stance of each company about sharing intellectual property during the pandemic is conflicted. AstraZeneca–Oxford and Moderna’s stances were positive. Oxford University recently pledged to make its COVID-19 intellectual property available through non-exclusive and royalty-free licenses [67]. Moderna also announced that while the pandemic continues, it would not enforce COVID-19 related patents [68]. There was no explicit statement from Sinopharm and Sinovac, but in May, the Chinese government expressed support for waiving intellectual property protections for novel coronavirus vaccines in a bid to help developing nations [69].

However, Pfizer/BioNTech and Janssen were strongly against waiving the TRIPS agreement [70,71]. The two companies argued that waiving intellectual property rights would create more problems. They stated that it could disrupt the flow of raw materials and disincentivize anyone else from taking a big risk [70]. There could also be a difference in manufacturing ability among companies and countries, making the quality of the vaccine hard to manage.

#### 4.3.5. Manufacturing Agreements

Table 6 compares how the vaccine developers were managing the manufacturing of their drugs. Manufacturing agreements have been made to cope with the shortage of drugs and to share the know-how of vaccine production around the globe. There are two types of manufacturing agreements: contract development and manufacturing organization (CDMO) and technology transfer. A CDMO is an organization or company that partners with the original vaccine developer and provides manufacturing services. Technology transfer involves moving an existing manufacturing site to another.

AstraZeneca–Oxford were the most active in entrusting production of their vaccines to middle-income countries (MICs) and LMIC. On the other hand, Pfizer/BioNTech, Janssen and Moderna had made most of their manufacturing agreements only with their trusted partners in developed countries. Pfizer/BioNTech had made 17 contracts with HICs out of a total of 21 contracts, Janssen had made 7 contracts with HICs out of a total of 9 contracts, and Moderna had made 10 contracts with HICs out of a total of 11 contracts.

Summary of findings in terms of Research and Development, Transparency and Accountability, and Product Delivery are shown in Table 7.

### 4.4. Scoring of the EUL-Listed Vaccine Developers

The scores of the EUL vaccine developers’ CSR activity during the COVID-19 are shown in Table 8. The determined ranking was AstraZeneca–Oxford (6 points), Sinovac (6 points), Janssen (5 points), Sinopharm (5 points), Pfizer/BioNTech (4 points), and Moderna (4 points), in order.

## 5. Discussion

### 5.1. Findings

#### 5.1.1. Research and Development

There was a rapid development of vaccines by pharmaceutical companies around the world, and novel technology, the mRNA vaccine, has been introduced by Pfizer/BioNTech and Moderna. This technological breakthrough has been supported by a tremendous amount of public funding, which increases the importance of the companies practicing CSR.

AstraZeneca–Oxford and Janssen pledged “no-profit” on the COVID-19 vaccines, whereas Pfizer/BioNTech and Moderna embraced “slight profits” [72]. Pfizer/BioNTech and Moderna exhibited extensive revenues from COVID-19 vaccine sales this year. The profits companies are generating from their COVID-19 vaccines can be seen as a reward for the risks they have undertaken in developing the products. However, the huge amounts of public money that have been committed could compensate the companies for the costs of development, manufacture, and distribution, substantially de-risking it. For instance, COVAX has invested extensively in companies to accelerate the development of vaccines.

The existence of indemnification clauses also helps de-risking the vaccine developers. Governments and international agencies such as WHO and COVAX have used indemnification clauses as a tool to facilitate the supply and development of vaccines. They ensure that the developers will have “no-fault” even if some injuries are caused by the vaccines [73]. There are similar clauses in the contracts with many other countries including the United States [74]. Even if missteps are made or rare adverse effects are found, the developers will not bear the risks. Eccleston-Turner et al. point out that this could represent a win–win situation for vaccine developers, being able to benefit regardless of the clinical success and regulatory approval of their vaccine candidates [75]. These public efforts made for the development of the COVID-19 vaccine lead us to question as to whether the large amounts of profits some vaccine developers are making are righteous.

#### 5.1.2. Transparency and Accountability

Another problem regarding development of the COVID-19 vaccine was the lack of public disclosure on the exact methods and results of clinical trials. This can raise doubts and potentially undermine public confidence in the vaccine. Improvements in the transparency and accountability of the vaccine development process are needed.

Pfizer/BioNTech and Moderna have had the clearest clinical trial results. However, the U.S. executives at both companies were reported to have had adopted or amended pre-scheduled stock trading plans just days before significant announcements regarding COVID-19 vaccines were made public [76,77]. Such activities can raise suspicions of pharmaceutical companies using important public health announcements to make personal gains.

Issues about the transparency of vaccine contracts also need more public attention. All stakeholders can benefit when information on prices, the number of products to be supplied, and timetables for delivery are made accessible to the public. Businesses can use the information to win contracts fairly [78], and civil society and journalists can monitor irregularities and investigate corruption. Legislators can scrutinize the details of a deal, reducing the risk of corruption or malfeasance. Governments can improve their understanding of procurement processes and make informed choices [79].

However, in the context of the COVID-19 pandemic, details of the contracts have rarely been revealed, and such benefits of transparent contracts are mostly lost. Even COVAX has not released a single contract, and has reportedly justified this by noting that it “could be detrimental to our future deals” because they “contain proprietary information” [80]. This argument is standard within the industry [81]. This reasoning, however, does not justify the complete lack of publication of contracts. The need to secure the secrecy of proprietary information can be achieved by publishing a redacted version of the contract, if the redactions explain that they cannot arbitrarily be abused [6].

#### 5.1.3. Product Delivery

The vaccine delivery side is the most problematic. The participation of pharmaceutical companies through COVAX, especially, was significantly low. Due to the deficiency of vaccines secured by COVAX, even lower-income countries were starting to purchase vaccines directly through a bilateral contract with the company. LICs and LMICs did not have enough vaccines, whereas some higher-income countries have secured enough COVID-19 vaccines to protect a population almost four times their size [82].

The limited manufacturing capacity is one of the main reasons for the deficiency in vaccines in LMICs, and it is thought that there will not be enough vaccine doses to protect the global population until 2023 or 2024 [83]. The limited supply of vaccines places suppliers, in this case, vaccine developers, in a very powerful position. In such a market, it is easier for most low-income countries to be excluded from the competition because of their low purchasing power. Their low bargaining power may also be the reason for the lack of a linear relationship between a country’s economic capability and the vaccine price, as found in our study. The secrecy of vaccine supply agreements further limits countries’ negotiating power, because it makes it difficult for countries to review the evidence and measure costs and benefits. This highlights our previous point, that transparency is essential in acquiring sufficient bargaining power for LMICs and increase vaccine distribution equality.

The commonly used policies for reducing prices, such as encouraging competition, generic substitution, and external and internal reference pricing, are also hard to apply in this situation because of the rapid pace of vaccine development and the lack of sufficient safety, efficacy, and pricing data [84]. Therefore, equitable pricing, or tiered pricing, becomes more important. Moon et al. have pointed out that although market competition is generally a more effective method to lower prices, tiered pricing can contribute to improved access in the short term when markets are small, highly uncertain, production capacity is limited, or has a time delay in overcoming barriers to competition [31]. The current market of COVID-19 broadly matches the explanation above: it is a ground where equitable pricing can be very influential.

However, we should consider the possible drawbacks of tiered pricing. Tiered pricing policies give considerable decision-making power to private firms, whose pricing decisions may not necessarily match with public interest, as we have observed in the COVID-19 pandemic. Another criticism is that there is no straightforward way to set tiered prices to achieve affordability [31]. Therefore, alternate strategies such as harnessing the power of competition and recognizing government responsibilities should be explored.

Furthermore, increasing the bargaining power of LMICs is crucial. COVAX is one of the existing solutions attempting to give LMICs sufficient capacity for negotiation; however, our study found that most contracts at present are bilateral, rather than via COVAX. There are many problems with bilateral contracts. First, the size of bilateral contracts is generally smaller than multilateral contracts, making a country’s negotiating ability relatively poor. In effect, all the vaccine prices determined by a bilateral contract are much higher than the price COVAX delivers (USD 3.2/dose). Second, there is a problem of transparency, and the public cannot know if the pharmaceutical company is supplying the vaccine according to the principle of equitable pricing. Third, the public image of pharmaceutical companies is diminished. It is important to keep in mind that the principle of CSR benefits both the public and the company. If all countries and firms sign contracts collectively via COVAX, rather than making bilateral contracts, they would be able to secure bargaining power, and companies would also be compensated sufficiently for their efforts. Therefore, we strongly recommend that the pharmaceutical industry increase engagement in COVAX.

The use of compulsory or voluntary licensing agreements is also an important strategy LMICs can use; therefore, the importance of waiving IP and the incorporation of LMICs in the production process grow stronger as the pandemic wears on. The main problem is that vaccine manufacturing, research, and development are too heavily concentrated in a small group of high- and middle-income countries. The world would be able to share the burden of the development of essential pharmaceuticals and vaccines through IP waivers, and it would reduce the barriers, particularly for LMICs, to give them opportunities to be medically and economically independent. Countries backing the IP waiver are not asking for charity, but for the right to develop and make their vaccines, free from the worry that they will be sued by patent-holders [85]. However, the EUL vaccine developer’s stance on waiving IP rights is also conflicted. Efforts in working together with LMICs for the manufacture of vaccines could be only seen by AstraZeneca–Oxford, Sinopharm, and Sinovac.

#### 5.1.4. Assessment of Vaccine Developer’s CSR and Suggestions

There has been a difference in companies’ existing CSR practice and their actions during the pandemic. According to the Access to Medicine Index [44], Janssen (3.76 points), Pfizer (3.65 points), and AstraZeneca (3.30 points) were ranked third, fourth, and seventh, respectively. The ranking of relatively small and new companies—Moderna, Sinovac, and Sinopharm—was not measured. The high rankings of Janssen and Pfizer by this index suggest that these two companies will demonstrate the most responsible behavior according to CSR in a pandemic situation. This is conflicted by the low CSR score Pfizer was attributed in our study; they were against sharing intellectual property, provided fewer supplies to LMICs, and displayed limited participation in COVAX. On the other hand, AstraZeneca, which ranked the lowest in the Access to Medicine Index (7th), demonstrated more compliance with CSR, making many more vaccine supply contracts and manufacturing agreements with LMICs relative to the other pharmaceuticals. We can conclude that there is a difference in the CSR behavior of companies in the special context of COVID-19.

An emphasis on social pressure may be the most effective way to push pharmaceutical companies toward CSR. In the past, Oxfam and Médecins Sans Frontières (MSF) joined forces to cut drug prices and promote access to antiviral medication for LMICs, and have witnessed particular success [86]. Due to their pressure and generic competition, the price of antiretroviral drugs has dropped by more than 99% over the last decade [87]. A pharmaceutical or biotechnology company could also take the lead on CSR to be a pioneer in their industry. Guidelines and rankings serve as a strong motivation for companies; therefore, we strongly recommend establishing guidelines and recommendations to facilitate CSR in pandemic situations.

### 5.2. Contributions and Implications

The strength of this study is that it comprehensively analyzed the pharmaceutical industry’s CSR activities using various sources, being the first to focus on the CSR of COVID-19 vaccine developers.

We have reinforced the extant literature on exploring CSR activities during crises or addressing the social responsibility of the pharmaceutical industry. Additionally, we have discussed the limitations of the current indexes such as the Access to Medicine Index and attempted to fill in the gaps. Thus, we have contributed to constructing a methodology of measuring companies’ CSR activities. Our study also helps to increase the understanding of the current inequality in the distribution of the COVID-19 vaccines. Our findings can help companies and relevant stakeholders to re-examine the role of business and the meaning of social responsibility in these difficult times of the pandemic, and thus help to improve the current inequalities.

Research on each country’s political strategy and international relations is, of course, important in understanding the issue of vaccine distribution. However, vaccine developers, especially multinational pharmaceutical companies, also play a critical role in vaccine distribution and manufacturing. Although discourse regarding the role of each country is relatively abundant, discussions concentrated on the vaccine developer’s role are insufficient. We expect our research to contribute to a more comprehensive understanding of COVID-19 vaccine inequality. More qualitative and quantitative analyses based on methods of multi-objective optimization [88] and attention to the role of pharmaceutical companies, and the relationship between pharmaceutical companies and the nation during a pandemic, are needed.

### 5.3. Limitations

There were several limitations to our study. First, we only focused on the current vaccine developers. As well as vaccines, other medical treatments are important in overcoming COVID-19, and some pharmaceutical companies not mentioned in this paper have practiced CSR in this matter. For example, AbbVie announced that they will not enforce exclusivity rights over the drugs lopinavir and ritonavir, not only for use on COVID-19, but for any use, anywhere in the world [89]. Merck also announced that they will make their intellectual property available related to the drug hydroxychloroquine to support broad access to the drug [90]. Therefore, to analyze the overall trend of the pharmaceutical industry, companies related to the production of medicine for the treatment of COVID-19, protective equipment for medical workers, and public health system strengthening should also be considered.

Second, the current problem of COVID-19 vaccine distribution is closely related to the international strategies and policies of each nation. For example, “vaccine nationalism”, referring to the actions of some developed countries that are adopting policies that prioritize their own public health needs at the expense of others [91], has negatively affected the equitable distribution of vaccines. Many developed countries have approached vaccine procurement with power politics and struggled to make bilateral Advance Purchase Agreements with vaccine developers, in trying to calm domestic public sentiment which has been aggravated by the pandemic [91]. A political perspective is needed to understand the whole scope of the problem, although we did not address it because it was out of the scope of this study.

Third, most of the data used in this paper were from secondary sources and insufficient. Internal information from companies, especially Sinopharm and Sinovac, was difficult to obtain. The analysis on published contracts was based on data extracted on 5 March 2021 by Transparency International [6]; therefore, it was limited for understanding the current situation. More information on contract details and vaccine price data is need for accurate analysis. Further research and updates should be made to investigate and analyze the internal policies of pharmaceutical companies in more detail.

## 6. Conclusions

The importance of CSR is indispensable for the pharmaceutical industry in the global COVID-19 crisis. The international community has recognized the importance of equity in the distribution of vaccines and has made some efforts, such as establishing COVAX, but further effort is needed. We evaluated the CSR of the vaccine developers in three categories—Research and Development, Transparency and Accountability, and Product Delivery, and have summarized points on which the industry could improve. The lack of the public disclosure of clinical data and vaccine supply agreements, lack of engagement though multilateral deals such as COVAX, and insufficient corporate policies (e.g., equitable pricing, IP strategy, manufacturing agreements) leading to inequality in the distribution of vaccines was highlighted. The scoring of vaccine developers indicated that the companies’ CSR practices have differed during the pandemic.

Our study contributes to the methodology of assessing the social responsibility of various pharmaceutical companies. We have also helped further the understanding of contemporary COVID-19 vaccine distribution inequality and propose that pharmaceutical companies re-examine their roles and social responsibilities. Further research on pharmaceutical companies’ behavior in response to global emergencies is needed. Global recommendations and agreements to facilitate CSR in pharmaceutical firms are warranted.

## Figures and Tables

**Table 1:** Effectiveness, storage, and manufacturing capacity of EUL COVID-19 vaccines.

	Pfizer/BioNTech	AstraZeneca–Oxford	Janssen	Moderna	Sinopharm	Sinovac
Effectiveness [43] ^1^	95%	62–90%	66%	95%	79–86%	50–92%
Storage ^2^	Ultra-cold (−60 to −80 °C)	Refrigeration (2~8 °C)	Refrigeration (2~8 °C)	Refrigeration (2 °C to 8 °C) for up to 30 days or frozen (−15 °C to −25 °C) for long-term storage	Refrigeration (2~8 °C)	Refrigeration (2~8 °C)
Manufacturing capacity in 2021 ^2^	3 billion doses per year	3 billion doses	1 billion doses per year	Up to 1 billion doses	Up to 1 billion doses Beijing/Sinopharm combined with Wuhan/Sinopharm vaccine	Up to 2 billion doses per year

Notes: ^1^ Preliminary phase III results, not yet peer-reviewed. Effectiveness data reported from BBC News [43] ^2^ Storage and Manufacturing capacity data are from the COVID-19 vaccine tracker [8].

**Table 3:** Transparency of clinical trials and published contracts of EUL COVID-19 vaccines.

Company	Clinical Trial Protocol Shared	Clinical Trial Results Shared (Peer Reviewed) ^1^	Published Contracts	
			Number of Published Contracts ^3^ (%)	Number of Released Vaccine Prices (%) ^4^
Pfizer/BioNTech	Yes	Phase I/II/III: 7 (3) Phase I/II: 3 (-)	3 (7.1)	8 (10.7)
AstraZeneca–Oxford	Yes	Phase I/II: 7 (3) Phase II/III: 4 (1) Phase III: 6 (-)	5 (7.9)	22 (31.4)
Janssen	Yes	Phase I/IIa: 2 (2) Phase III: 1 (-)	1 (10.0)	3 (16.7)
Moderna	Yes	Phase I: 3 (3) Phase III: 3 (1)	1 (9.1)	7 (24.1)
Sinopharm	No	Phase I/II: 2 (-) Phase III: 3 (-)	-	7 (14.9)
Sinovac	Yes	Phase I/II: 3 (-) Phase III: 6 (-)	-	9 (22.5)
Overall ^2^	- Clinical trial protocols had been published for 12% of trials	- Results from 45% of these trials had been announced - 41% of the current vaccines had provided only top-level results via a press release or press conference, full data not made available for media scrutiny or academic review	- Only 6% of the concluded agreements were formally published on 5th March 2021 - Of the total 279 vaccine supply agreements made until August 2021, only 56 (19%) vaccine prices are known	

Notes: Data were acquired from Transparency International [6] (published May 2021, data extraction performed until 5 March 2021), the UNICEF COVID-19 Vaccine Market Dashboard [7] and the COVID-19 vaccine tracker [8]. “-” means that the numbers were not announced publicly. ^1^ The number indicates how many times the clinical trial results had been shared. The number in the brackets indicates how many of the clinical trial results had been peer reviewed. For example, the Pfizer/BioNTech vaccine had shared their Phase I/II/III results 6 times, and 2 of them were peer-reviewed. This column presents reorganized data from Transparency International [6] ^2^ Summary data source is Transparency International [6]. ^3^ Number of public contracts were found by Transparency International [6]. The report was written based on the UNICEF data up to 5 March 2021; therefore, we only integrated data up to 5 March 2021 for the calculation. The total number of announced contracts by each company was divided to acquire the percentage of published contracts. The percentage is shown inside the brackets under the numbers. ^4^ We further assess how much of the vaccine prices were revealed. The number of released vaccine contracts is based on the UNICEF COVID-19 Vaccine Market Dashboard [7] up to August 2021. The total number of announced contracts by each company was divided and is shown in percentages to show the scope of vaccine price transparency.

**Table 4:** Vaccine supply agreements made by the vaccine developers, sorted by type of agreement and economy level.

		Pfizer/BioNTech	AstraZeneca–Oxford	Janssen	Moderna	Sinopharm	Sinovac	Total
Total Secured Doses (Millions)		4040	3434	923	1859	418	645	11,319
Number of doses by type of agreement (millions)	multilateral (%)	1544 (38.2)	1020 (29.7)	657 (71.1)	960 (51.6)	60 (14.3)	50 (7.7)	4291 (37.9)
	bilateral ^1^ (%)	2496 (61.8)	2414 (70.3)	266 (28.9)	899 (48.4)	358 (85.6)	595 (92.2)	7029 (62.1)
Number of doses by country income level (millions) ^2^	COVAX ^3^ (%)	540 ^5^ (13.4)	720 (21.0)	200 (21.7)	500 (26.9)	60 (14.3)	50 (7.7)	2070 (18.3)
	HIC (%)	2701 (66.9)	1025 (29.8)	417 (45.1)	1297 (69.7)	11 (2.5)	169 (26.2)	5619 (49.6)
	UMIC (%)	619 (15.3)	455 (13.3)	63 (6.8)	37 (2.0)	80 (19.2)	198 (30.7)	1453 (12.8)
	LMIC (%)	179 (4.4)	1216 (35.4)	243 (26.4)	25 (1.3)	256 (61.3)	227 (35.2)	2147 (19.0)
	LIC (%)	0	18 (0.5)	0	0	11 (2.7)	0.3 (0.1)	11 (0.1)
Total number of agreements		75	70	18	29	47	40	279
Number of agreements by type of agreement ^4^	multilateral (%)	4	3	4	4	1	1	17
	bilateral (%)	71	67	14	25	46	39	262
Number of agreements by country income level	COVAX	2	2	1	1	1	1	8
	HIC	33 (30 + 3) *	20 (19 + 1)	8 (6 + 2)	20	5	5	7
	UMIC	24	19	4	5	19	19	90
	LMIC	16	28	5 (4 + 1)	3	20	14	81
	LIC	0	1	0	0	2	1	4

Notes: All data were calculated from data retrieved from UNICEF, COVID-19 Vaccine Market Dashboard [7] until August 2021. ^1^ Bilateral includes bilateral deals, private purchase, and licensing deals made between two countries. ^2^ The multilateral deal made with the European Commission was calculated as contracts with HICs as the average GDP per capita of HIC countries (USD, 43,834) fitting the definition of HICs by the World Bank [62]. The number of the included multilateral deals are shown inside the column (*); for example, Pfizer had made three deals with the European Commission, and thus is shown as (30 + 3) * in the above table. The multilateral deal made with the African Union was calculated as contracts with LMICs, as the average GDP per capita (USD 2406) of the African Union countries, fitting the definition of LMIC by World Bank. LICs are defined as those with a GNI per capita, calculated using the World Bank Atlas method, of USD 1045 or less in 2020; LMICs are those with a GNI per capita between USD 1046 and USD 4095; UMICs are those with a GNI per capita between USD 4096 and USD 12,695; HIC are those with a GNI per capita of USD 12,696 or more. ^3^ Contracts made with COVAX was not included in classification by country due to its difficulty of including it into a specific country economy level. ^4^ Number of publicly announced supply agreements. If the same recipient has made contracts with the developer several times, it was not excluded but all counted ^5^ Pfizer—“COVAX” includes the bilateral contract the USA made with Pfizer for donations to the COVAX facility (500 million doses).

**Table 5:** Average vaccine prices and correlation between GDP per capita and vaccine prices (USD).

(USD).	Pfizer/BioNTech	AstraZeneca–Oxford	Janssen	Moderna	Sinopharm	Sinovac	Average
Average	14	5	9	25	24	18	13
COVAX	3	3	3	3	3	3	3
Minimum	7	2	8	15	15	10	N/A
HIC Average	19	4	9	25	36	N/A	16
UMIC Average	11	4	N/A^2^	25	24	22	15
LMIC Average	7	5	10	N/A	17	15	9
Maximum	23	13	10	37	36	32	N/A
Coefficient	0.82	−0.26	−0.02	−0.13	0.78	0.67	0.22
# ^1^	8	22	3	7	7	9	N/A

Note: All data were calculated from data retrieved from UNICEF, COVID-19 Vaccine Market Dashboard [7] up to August 2021. ^1^ Number of available vaccine prices in the COVID-19 Vaccine Market Dashboard. ^2^ Indicated as N/A when there were no vaccine prices available for calculating the average.

**Table 6:** Number of manufacturing agreements of EUL vaccine manufacturers.

	Contract Development and Manufacturing Organization (CDMO)			Technology Transfer		
Company	HIC	UMIC, LMIC	Total	HIC	UMIC, LMIC	Total
Pfizer/BioNTech	16	1	17	1	3	4
AstraZeneca–Oxford	16	-	16	2	7	9
Janssen	7	1	8	-	1	1
Moderna	10	1	11	-	-	-
Sinopharm	-	-	-	1	4	5
Sinovac	-	-	-	-	7	7

Note: Calculated by data retrieved from UNICEF, COVID-19 Vaccine Market Dashboard [7] up to August 2021. “-” means that the numbers were not announced publicly.

**Table 7:** Summary of findings.

Category	Indicators	Good	Need Improvements
Research and Development (4.1)	Effectiveness (4.1.1)	- mRNA vaccines (Pfizer/BioNTech, Moderna) had highest effectiveness.	- Other vaccines using traditional techniques showed large variations in effectiveness; - There was a large range in the reported efficacy of Sinovac’s vaccine.
	Storage ^1^ (4.1.1)	- Traditional vaccines only need to be refrigerated, more practical to reach remote parts of the world.	- Storage conditions of mRNA vaccines are comparatively delicate, tricky to survive through the cold chain.
	Manufacturing capacity ^2^ (4.1.1)	- Pfizer/BioNTech and AstraZeneca–Oxford had the highest manufacturing capacity (3.0 billion each).	- Manufacturing capacity announced by the companies may be overstated.
	Funding (4.1.2)	- There was a large amount of public effort and involvement in the development of vaccines.	- The exact amount of public funding is not clear, especially in the cases of Sinopharm and Sinovac.
	Profits (4.1.2)	- AstraZeneca–Oxford and Janssen pledged to not take profit on the COVID-19 vaccine.	- Lack of data for Sinopharm and Sinovac on revenues from vaccine sales and stance on profit from the COVID-19 vaccine.
Transparency and Accountability (4.2)	Clinical trials (4.2.1)	- Pfizer/BioNTech, AstraZeneca–Oxford, Janssen, and Moderna had announced their clinical trial protocol; - Pfizer/BioNTech and Moderna had the most clear and transparent clinical trial results.	- There is a significant lack of public disclosure of clinical data; - Sinopharm’s clinical trial protocols have not been shared; - Criticism of Janssen and AstraZeneca’s clinical studies.
	Vaccine Contracts (4.2.2)		- Most of the vaccine supply agreements were bilateral deals; - The details of the contracts were rarely revealed; - An overall lack of price data (of the total 279 vaccine supply agreements, only 56 vaccine prices are known).
Product Delivery (4.3)	COVAX % (4.3.1)	- Moderna was supplying their products via COVAX with the highest rate (27.63%), followed by Sinopharm (20.62%) and Janssen (19.02%).	- Significant lack of engagement through COVAX; - Pfizer/BioNTech, Sinopharm, and Sinovac’s participation through COVAX is limited (under 15%).
	Distribution to Lower Income Countries (4.3.2)		- 49.64% of total vaccine supply were being given to HICs; - Shortage of supplies for LMICs and LICs.
	Equitable Pricing (4.3.3)	- Pfizer/BioNTech has the highest correlation coefficient between GDP per capita and vaccine price (0.82); - Sinopharm and Sinovac also providing vaccines at more affordable price to lower-income countries; - AstraZeneca–Oxford has the lowest vaccine price (average 4.77 USD/dose).	- AstraZeneca–Oxford does not seem to have made their products cheaper to lower income countries; - Only three price data values are available for Janssen.
	Intellectual Property Strategy (4.3.4)	- AstraZeneca–Oxford and Moderna have approved the waiving of intellectual property while the pandemic continues; - There was no explicit statement from Sinopharm and Sinovac, but in May, the Chinese government expressed support for waiving intellectual property protections.	- Pfizer/BioNTech and Janssen strongly against waiving TRIPS agreement.
	Manufacturing Agreements (4.3.5)	- AstraZeneca–Oxford were most active in entrusting production of their vaccines to UMICs and LMICs; - Sinopharm and Sinovac were actively working with foreign companies.	- Pfizer, Janssen, and Moderna had most of their manufacturing contracts only within their partners in developed countries.

Note: Discussion on Storage ^1^ conditions and Manufacturing capacity ^2^ was included to describe the current vaccine distribution situation. However, they have not been included in the scoring of CSR practices because it could be considered out of the scope of CSR.

**Table 8:** Scoring of the EUL vaccine developers.

Category	Indicators	Pfizer/BioNTech	AstraZeneca–Oxford	Janssen	Moderna	Sinopharm	Sinovac
Research and Development	Effectiveness	1			1		
	Profits		1	1			
Transparency and Accountability	Clinical trials	1	1	1	1		1
	Vaccine Contracts		1		1		1
Product Delivery	COVAX %			1	1	1	
	Distribution to Lower Income Countries		1	1		1	1
	Equitable Pricing	1				1	1
	Intellectual Property Strategy		1	1		1	1
	Manufacturing Agreements	1	1			1	1
Total		4	6	5	4	5	6
Ranking		3	1	2	3	2	1

## Data Availability

Not applicable.

## Supplementary Materials

The following are available online at https://www.mdpi.com/article/10.3390/vaccines9101183/s1, Table S1: List of EUL COVID-19 vaccines, Table S2: Categories and indicators measured for the Access to Medicine Index, Table S3: Source of Materials.
