# Supplementary material for: Pharmaceutical Industry’s Engagement in the Global Equitable Distribution of COVID-19 Vaccines: Corporate Social Responsibility of EUL Vaccine Developers
Source: Vaccines (Basel). 2021 Oct 15;9(10):1183. doi: 10.3390/vaccines9101183 (PMC8539183; doi:10.3390/vaccines9101183)
Supplement: Supplementary file 1 [file vaccines-09-01183-s001.zip › vaccines-1373329-supplementary.pdf]

Table S1. List of EUL listed COVID-19 vaccines

No.	Vaccine Developer	Name of Vaccine	Type of Vaccine	Emergency Use Listing (EUL) listed
1	Pfizer/BioNTEcch	BNT162b2/COMIRNATY Tozinameran (INN)	RNA	31/12/2020
2	AstraZeneca-Oxford	AZD1222 Vaxzevria	Non-Replicating Viral Vector	16/04/2021
		Covishield (ChAdOx1_nCoV-19) [1]		15/02/2021
3	Janssen	Ad26.COV2.S	Non-Replicating Viral Vector	12/03/2021
4	Moderna	mRNA-1273	RNA	30/03/2021
5	Sinopharm	SARS-CoV-2 Vaccine (Vero Cell), Inactivated (InCoV)	Inactivated	07/05/2021
6	Sinovac	COVID-19 Vaccine (Vero Cell), Inactivated/Coronavac ™	Inactivated	01/06/2021

[1] Notes: the Covishield Vaccine is listed as a separate developer (Serum Institute of India) in the status of COVID-19 Vaccines within WHO EUL/PQ evaluation process [43]. In this study, we analyzed AZD1222 Vaxvria and Covishield vaccine together as a vaccine made by a same developer: AstraZeneca-Oxford.

Table S2. Categories and indicators measured for the Access to Medicine Index.

Access to Medicine Index		Modified Categories for this study	
**Technical Areas**	**Priority Topics**	**Technical Areas**	**Indicators**
Governance of Access	Responsible business practices<br>Governance and Strategy		
Research and Development	Access planning	Research and Development (3.1)	
	Product development		Effectiveness (3.1.1)
	Building R&D Capacity		Funding (3.1.2)<br>Profit Generation (3.1.2)
		Transparency and Accountability (3.2)	Clinical Trials (3.2.1)<br>Vaccine Contracts (3.2.2)
Product Delivery		Product Delivery (3.3)	COVAX % (3.3.1)<br>LMIC % (3.3.2)
	Equitable access strategies		Equitable Pricing (3.3.3)
	Intellectual property Strategy		Intellectual Property Strategy (3.3.4)
	Quality and supply		
	Licensing quality		
	Product donations		
	Registration		
	Inclusive business models		
	Local manufacturing		Manufacturing Agreements (3.3.5)
	Health systems strengthening		

Table S3. Source of Materials

Result	Source	Source type	Search methodology	Date Limit for search (Last Day Accessed)	Date Published
4.1	[47] Ruchir Agarwal	Report (IMF)			May 19, 2021
	[44] Tan, Y. Covid: What do we know about China's coronavirus vaccines?	News Article (BBC News)	Google search for characteristic of COVID-19 vaccines	Jan 2020 ~ Aug 2021 (15 Sep 2021)	14 January, 2021
	[8] COVID-19 vaccine tracker	Internet Database (Lancet Global Health)			May 2021
	[48] COVID-19 Vaccines R&D Investments	Report (The Knowledge Network on Innovation and Access to Medicines)	Integrated data on funding companies received from report	Jan 2020 ~ Aug 2021 (23 Sep 2021)	May 2021
	[49] Pfizer, Pfizer reports strong second-quarter 2021 results	Financial Report (Pfizer)	Financial report from official company website or SEC.gov	Jan 2021 ~ Aug 2021 (9 Oct 2021)	July 4, 2021
	[56] Pfizer, Pfizer reports strong First-quarter 2021 Results.				April 4, 2021
	[57] AstraZeneca, H1 2021 results	Financial Report (AstraZeneca)			July 29, 2021
	[58] Johnson&Johnson Reports Q2 2021 results	Financial Report (Johnson&Johnson)			July 21, 2021
	[59] Johnson&Johnson Reports 2021 first-quarter results				April 20, 2021
	[60] Moderna,10-Q Quarterly Report(Q1)	Financial Report (Moderna)			May 6, 2021
	[61] Moderna, 10-Q Quarterly Report(Q2)				Aug 5, 2021
	[50] Sinopharm, 2021 Interim Report	Financial Report (Sinopharm)			Oct 9, 2021
	[51] Parton, J. Covid Shot Makers to Share In Up to $190 Billion Sales Bonaza	News Article (Bloomberg)	Google search on stance of companies on profit by COVID-19 vaccines	Jan 2020 ~ Aug 2021 (15 Sep 2021)	May 26, 2021
	[52] Shapiro, E. Pfizer CEO Albert Bourla Raises Expectations That	News Article (TIME)			July 9, 2020

	the Pharmaceutical Giant Can Deliver a COVID-19 Vaccine by Fall.				
	[53] Dunn, A. The CEO of the buzzy biotech that's working on a potential coronavirus vaccine just pledged he won't set a high price for the shot	News Article (Business Insider)			May 5, 2020
	[54] Douoguih, M. Testimony	Statement submitted to U.S. House of Representatives			Oct 10, 2020
	[55] Pangalos, M. Pathway to a Vaccine: Efforts to Develop a Safe, Effective and Accessible COVID-19 Vaccine				July 21, 2020
4.2	[6] Transparency International	Report	Reorganized data from the report and internet databases to acquire results	Jan 2020 ~ Aug 2021 (23 Sep 2021)	May 2021
	[7] UNICEF COVID-19 Vaccine Market Dashboard	Internet Database			Updated monthly
	[8] COVID-19 vaccine tracker	Internet Database (Lancet Global Health)			May 2021
	[62] Roberts, M. Oxford/AstraZeneca Covid vaccine 'dose error' explained.	News Article (BBC)	Reference cited on Report[6]	(23 Sep 2021)	Nov 27, 2020
4.3	[7] UNICEF, COVID-19 Vaccine Market Dashboard	Internet Database	Used data until August 2021 to calculate results	Jan 2020 ~ Aug 2021 (15 Sep 2021)	Updated monthly
	[68] Innovation, O.U. Expedited access for COVID-19 related IP.	Company Website (AstraZeneca-Oxford)	Google search on announcements of companies on IP rights	Jan 2020 ~ Aug 2021 (15 Sep 2021)	
	[69] Loftus, P. Moderna Vows to Not Enforce Covid-19 Vaccine Patents During Pandemic	News Article (The Wall Street Journal)			Oct 8, 2020
	[70] McCarthy, S. China backs IP waiver for coronavirus vaccines	News Article (South China Morning Post)			May 17, 2021

	[71] Bourla, A. An open letter from Pfizer Chairman and CEO Albert Bourla	Press Release (Pfizer)		Accessed on 15 July 2021
	[72] Eakin, B. J&J's Chief Patent Atty Says COVID IP Waiver Won't Work.	News Article (Law 360)		April 22, 2021
